# Supplementary material for: Data on strategic change on employees’ behavioural attitude and firm performance of selected manufacturing firms in Nigeria
Source: Data Brief. 2018 Apr 14;18:1551–5. doi: 10.1016/j.dib.2018.04.032 (PMC5997772; doi:10.1016/j.dib.2018.04.032)
Supplement: Supplementary file 2 — Supplementary material [file mmc2.docx]

**Data on Strategic Change on Employees’ Behavioural Attitude and Firm Performance of Selected Manufacturing Firms in Nigeria**

**Chinyerem Adeniji; Covenant University**

**Olufemi Adeyeye; Covenant University**

**Oluwole Iyiola; Covenant University**

**Maxwell Olokundun; Covenant University**

**Deborah Motilewa; Covenant University**

**Stephen Ibidunni; Covenant University**

**Mosunmola Akinbode; Covenant University**

**Introduction**

*In today’s business environment, organizations must continually and constantly reinvent themselves to stay relevant because they conduct operations in workplaces that are characterized by steady competition and erratic change. Most studies show that organizational improvement cannot occur without strategic changes directed to yield a difference in performance. Thus, improving performance requires the consideration of change-related policies and individuals’ dispositions relevant to change. Strategic change as perceived by many authors requires qualitative changes and not simple continuous and usual changes. Strategic change must be aligned to the mission, and purpose of an organization. Employees’ attitudes towards change strongly relates to their attitudes about their employer and changes at their organization because organizations continually commence new programs of organizational change, these ongoing and seemingly endless efforts put a lot of burden not only on organizations but also on individuals. Researchers highlight the challenges to strategic change as; poor organizational management and culture, increased technology installation, organizational structure, strong competition and employee issues. Attitudes toward strategic change are the feelings employees have toward different internal policies of the organization. Many investigations suggest that it is reasonable to expect employees to react to strategic change efforts since the process of change involves going from the known to the unknown. Consequently, it can be a very unpleasant experience for employees thus this article presents data in this regard*

Keywords: Strategic change; Employee Behavioural attitude; Firm performance; Manufacturing Companies; Nigeria

**Specification Table**

| **Subject area** | Business, Management |
| --- | --- |
| **More Specific Subject Area:** | Business Administration |
| **Type of Data** | Table. Figures |
| **How Data was Acquired** | Researcher-made questionnaire analysis |
| **Data format** | Raw, analyzed, Descriptive and Inferential statistical data |
| **Experimental Factors** | Sample consisted of employees of manufacturing companies in Nigeria. The researcher-made questionnaire which contained data strategic change on employees’ behavioural attitude and firm performance were completed. |
| **Experimental features** | Change strategy is a major factor endangering firm performance particularly in the manufacturing sector. |
| **Data source location** | South west Nigeria |
| **Data Accessibility** | Data is included in this article |

**Value of Data**

Findings reveal that a unit increase in strategic change will lead to an increase in employees’ behavioural attitude and firm performance showing that employees have a high degree of continuance commitment during the change.

The results could be seen as positive, not only to the behaviours, but also to the change climate in general. It could be assumed that the more employees perceive themselves to have a high choice in initiating and regulating actions, the more they perceive themselves able to impact the change.

This study indicates that increased competition aligned with internal policies positively affects employees’ attitude to organizational change

**1. Data**

The Figure and table below shows the predictor importance of strategic change on employees’ behavioural attitude and performance of selected manufacturing firms.

**Predictor Importance of strategic change on employees’ behavioural attitude and performance of Sampled Firms**


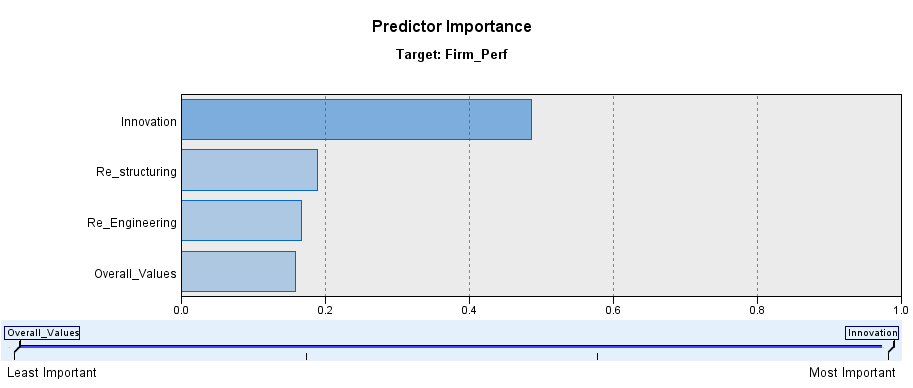


Figure 1: Predictor Importance of strategic change on firm performance

Source: Researcher‘s Field Survey Result (2017).

**Table 1: Importance of the Variables and as it is equal to 1**

| **Nodes** |  | **Importance** |
| --- | --- | --- |
| Innovation | Innovation | 0.49 |
| Restructuring | Restructuring | 0.19 |
| Re-engineering | Re-engineering | 0.17 |
| Overall Values | Overall Values | 0.16 |

Figure 1 and Table 1 above predicts the importance of the construct for independent variables on the dependent variables. In other words, it can be depicted that the construct with the least importance to firm performance is change in values while innovation and restructuring become the most important predictor of strategic change. This implies that change in firm performance could be as a result of the drastic change in the strategy and internal policies of the firms. However, to assess the coefficient (significant effects) level, regression analysis was adopted as presented in the table below. The level of significance below 0.05 shows the confidence of level of 95%. Therefore, under such circumstance, we reject the null (H_0_) hypothesis once P-value is less than or equals to 0.05 while we accept the alternate (H_1_) hypothesis. The regression analysis, specifically, regression and analysis of variance were employed to test the hypothesis because all the data are combination of ordinal and nominal data. This was used to examine the predictive capabilities of strategic change on employees’ behavioural attitude and performance of selected manufacturing firms.

| **Table 2: MODEL SUMMARY** | | | | | | | | | |
| --- | --- | --- | --- | --- | --- | --- | --- | --- | --- |
| Model | R | R Square | Adjusted R Square | Std. Error of the Estimate | Change Statistics | | | | |
|  |  |  |  |  | R Square Change | F Change | df1 | df2 | Significant F Change |
| 1 | .645a | .416 | .415 | .58224 | .416 | 300.221 | 1 | 421 | .000 |
| 2 | .661b | .437 | .434 | .57274 | .020 | 15.091 | 1 | 420 | .000 |
| a. Predictors: (Constant), CHS | | | | | | | | | |
| b. Predictors: (Constant), CHS, BAA  Source: Researcher’s Field Survey, 2017 | | | | | | | | | |

Table 2 above tested the effect of strategic change on employees’ behavioural attitude and performance of selected manufacturing firms. In the first step, the effect of strategic change on the performance of selected manufacturing firms was tested. The R-Square value is the degree of variation of the dependent variable which can be predicted by the independent variable. The analysis revealed that strategic change accounted for 41.6% variance in firm performance of selected manufacturing firms (R^2^ = .41.6, df (1, 421) = 300.221, p ˂ .05). In the second step, the mediating role of employees’ behavioural attitude was examined. The analysis showed that employees’ behavioural attitude was able to explain 43.2% variance in firm performance over and beyond the effects of strategic change (R^2^ = .437, df (2, 420) = 162.680, p ˂ 0.05). The significance of the F-change was assessed and it was significant (0.000) as shown in the table below:

| **Table 3: ANALYSIS OF VARIANCE** | | | | | | |
| --- | --- | --- | --- | --- | --- | --- |
| Model | | Sum of Squares | df | Mean  Square | F | Sig. |
| 1 | Regression | 101.777 | 1 | 101.777 | 300.221 | .000b |
|  | Residual | 142.722 | 421 | .339 |  |  |
|  | Total | 244.499 | 422 |  |  |  |
| 2 | Regression | 106.727 | 2 | 53.364 | 162.680 | .000c |
|  | Residual | 137.772 | 420 | .328 |  |  |
|  | Total | 244.499 | 422 |  |  |  |
| a. Dependent Variable: Firm Performance | | | | | | |
| b. Predictors: (Constant), CHS | | | | | | |
| c. Predictors: (Constant), CHS, BAA  Source: Researcher’s Field Survey, 2017 | | | | | | |

Table 3 above shows the results of the two models. The first model showed the effect of strategic change on the performance of selected manufacturing firms. The F-value is calculated as the Mean Square Regression (101.777) divided by the Mean Square Residual (0.339), yielding F= 300.221. From this result, model 1 in the table is statistically significant (Sig =.000). The second model examined the effect of strategic change on employees’ behavioural attitude and performance of selected manufacturing firms. The F-value is calculated as the Mean Square Regression (53.364) divided by the Mean Square Residual (0.328), yielding F= 162.680 at an acceptable significant level of .000.

| **Table 4: COEFFICIENTS** | | | | | | | | | | |
| --- | --- | --- | --- | --- | --- | --- | --- | --- | --- | --- |
| Model | | Unstandardized Coefficients | | Standardized Coefficients | t | Sig. | 95.0% Confidence Interval for B | | Collinearity Statistics | |
|  |  | B | Std. Error | Beta |  |  | Lower Bound | Upper Bound | Tolerance | VIF |
| 1 | (Constant) | 1.939 | .119 |  | 16.258 | .000 | 1.705 | 2.174 |  |  |
|  | CHS | .526 | .030 | .645 | 17.327 | .000 | .466 | .585 | 1.000 | 1.000 |
| 2 | (Constant) | 1.588 | .148 |  | 10.716 | .000 | 1.297 | 1.879 |  |  |
|  | CHS | .467 | .033 | .573 | 13.947 | .000 | .401 | .533 | .795 | 1.258 |
|  | BAA | .152 | .039 | .160 | 3.885 | .010 | .075 | .229 | .795 | 1.258 |
| a. Dependent Variable: Firm Performance | | | | | | | | | | |

Based on the results in Table 2, Table 4 above revealed the contributions of strategic change to employees’ behavioural attitude and firms’ performance and their levels of significance. (Change in strategy; β = .573; t= 13.947; p < .05, employees’ behavioural attitude; β = .160; t= 3.885; p < .05).

**2. Experimental Design, Materials and Methods**

Data was gathered from employees’ in selected manufacturing companies with the aid of a researcher- made questionnaire based on the works of [1] [2], [3], [4]. [5]. The population of the respondents was made up of 6,998 employees. The questionnaire was self-administered to the respondents who willingly filled the research questionnaire Survey research design was adopted for this study where data was collected from a sample size of 600 employees from the three tiers of management of three manufacturing firms in Nigeria namely Cadbury, Plc, Unilever Plc and Seven-up Nigeria, Lagos State to determine the effect of strategic change on employees’ behavioural attitude of employees’ and organisational performance. The collected data were coded and entered into SPSS version 22. Data analysis was performed; using inferential statistical tests which involved Hierarchical Regression analysis.

Ethical Considerations

The researchers ensured that the respondents were well informed about the goal of the research and they were kept abreast with the research regime. Respondents were given the opportunity to stay anonymous and their responses were treated confidentially. Permission was sought from the relevant authorities in the organisations before the distribution of the copies of questionnaire.

**Acknowledgement**

The authors wish to acknowledge the management of Covenant University for providing full sponsorship for this research work

**References**

[1] Agboola, G. M. (2014). Employee attitude towards attributes of technological change as a determinant of organizational performance in Nigerian commercial banks. An unpublished Thesis Submitted to Covenant University in Partial Fulfilment of the Requirements for the Award of Doctor of Philosophy Degree (Ph.D.) in Business Administration.

[2][Ibidunni, S.](https://www.scopus.com/authid/detail.uri?authorId=57063238100&amp;eid=2-s2.0-84954478999), [Osibanjo, O.](https://www.scopus.com/authid/detail.uri?authorId=56580486600&amp;eid=2-s2.0-84954478999), [Adeniji, A.](https://www.scopus.com/authid/detail.uri?authorId=56629637700&amp;eid=2-s2.0-84954478999), [Salau, O.P.](https://www.scopus.com/authid/detail.uri?authorId=57063088800&amp;eid=2-s2.0-84954478999), [Falola, H.](https://www.scopus.com/authid/detail.uri?authorId=57063424500&amp;eid=2-s2.0-84954478999) (2016) Talent retention and organizational performance: A competitive positioning in Nigerian banking sector(Review) [*Periodica Polytechnica Social and Management Sciences*](https://www.scopus.com/sourceid/22363?origin=recordpage), 24(1), 1-13

[3] [Olufemi, A.J.](https://www.scopus.com/authid/detail.uri?authorId=35077407800&amp;eid=2-s2.0-84973174861), [Olaleke, O.](https://www.scopus.com/authid/detail.uri?authorId=56415044200&amp;eid=2-s2.0-84973174861), [Augusta, A.](https://www.scopus.com/authid/detail.uri?authorId=57200185724&amp;eid=2-s2.0-84973174861), [Maxwell, O.](https://www.scopus.com/authid/detail.uri?authorId=57163916200&amp;eid=2-s2.0-84973174861), [Fred, I.](https://www.scopus.com/authid/detail.uri?authorId=57189574436&amp;eid=2-s2.0-84973174861) (2016). Inventory Control and Performance of Manufacturing Firms. Journal of Engineering and Applied Sciences 11(2), 199-203.

[4]Osibanjo, O.A. [Salau, O.P.](https://www.scopus.com/authid/detail.uri?authorId=57063088800&amp;eid=2-s2.0-84992448362) [Falola, H.O.](https://www.scopus.com/authid/detail.uri?authorId=57063424500&amp;eid=2-s2.0-84992448362) [Oyewunmi, A.E.](https://www.scopus.com/authid/detail.uri?authorId=57189379926&amp;eid=2-s2.0-84992448362) (2016) Workplace stress: Implications for organizational performance in a Nigerian public University. [*Business: Theory and Practice*](https://www.scopus.com/sourceid/1000147123?origin=recordpage) 17(3) 261-269.

## [5][Worlu, R.](https://www.scopus.com/authid/detail.uri?authorId=56299632400&amp;eid=2-s2.0-84960434246), [Olokundun, M.](https://www.scopus.com/authid/detail.uri?authorId=57163916200&amp;eid=2-s2.0-84960434246), [Akinbode, M.](https://www.scopus.com/authid/detail.uri?authorId=56331362500&amp;eid=2-s2.0-84960434246), [Augusta, A.](https://www.scopus.com/authid/detail.uri?authorId=56299475300&amp;eid=2-s2.0-84960434246), [Inelo, F.](https://www.scopus.com/authid/detail.uri?authorId=57189574436&amp;eid=2-s2.0-84960434246) (2016). Stress Arising From Work Roles and Impediments to Organizational Performance. *Research Journal of Medical Sciences,* 10(1), 1-5.
